# p-mTOR, p-ERK and PTEN Expression in Tumor Biopsies and Organoids as Predictive Biomarkers for Patients with HPV Negative Head and Neck Cancer

**DOI:** 10.1007/s12105-023-01576-4

**Published:** 2023-07-24

**Authors:** W. W. B. de Kort, E. J. de Ruiter, W. E. Haakma, E. Driehuis, L. A. Devriese, R. J. J. van Es, S. M. Willems

**Affiliations:** 1 Department of Pathology, University Medical Center Utrecht, 3584 CX Utrecht, The Netherlands; 2 Department of Oral and Maxillofacial Surgery, University Medical Center Utrecht, 3584 CX Utrecht, The Netherlands; 3 Oncode Institute, Hubrecht Institute, Royal Netherlands Academy of Arts and Sciences (KNAW) and University Medical Center Utrecht, 3584 CT Utrecht, The Netherlands; 4 Department of Medical Oncology, University Medical Center Utrecht, Utrecht, The Netherlands; 5 Department of Head and Neck Surgical Oncology, Utrecht Cancer Center, University Medical Center Utrecht, 3584 CX Utrecht, The Netherlands; 6 Department of Pathology, University Medical Center Groningen, 9713 GZ Groningen, The Netherlands

**Keywords:** HNSCC, Head and Neck Squamous Cell Carcinoma, mTOR, ERK, PTEN, Biomarkers

## Abstract

**Background:**

Survival rates of head and neck squamous cell carcinoma (HNSCC) have only marginally improved in the last decades. Hence there is a need for predictive biomarkers for long-time survival that can help to guide treatment decisions and might lead to the development of new therapies. The phosphatidylinositol 3-kinase (PI3K)/AKT/mTOR signaling pathway is the most frequently altered pathway in HNSCC, genes are often mutated, amplificated and overexpressed causing aberrant signaling affecting cell growth and differentiation. Numerous genetic alterations of upstream and downstream factors have currently been clarified. However, their predictive value has yet to be established. Therefore we assess the predictive value of p-mTOR, p-ERK and PTEN expression.

**Methods:**

Tissue microarrays (TMA’s) of HPV-negative patients with oropharyngeal (*n* = 48), hypopharyngeal (*n* = 16) or laryngeal (*n* = 13) SCC, treated with primary chemoradiation (cisplatin/carboplatin/cetuximab and radiotherapy), were histologically stained for p-mTOR, PTEN and p-ERK. Expression was correlated to overall survival (OS), disease free survival (DFS) and locoregional control (LRC). Also p-mTOR was histologically stained in a separate cohort of HNSCC organoids (*n* = 8) and correlated to mTOR-inhibitor everolimus response.

**Results:**

High p-mTOR expression correlated significantly with worse OS in multivariate analysis in the whole patient cohort [Hazar Ratio (HR) 1.06, 95%CI 1.01–1.11, *p* = 0.03] and in the cisplatin/carboplatin group with both worse OS (HR 1.09, 95%CI 1.02–1.16, *p* = 0.02) and DFS (HR 1.06, 95%CI 1.00–1.12, *p* = 0,04). p-ERK expression correlated significantly with DFS in univariate analysis in the whole patient cohort (HR 1.03, 95%CI 1.00–1.05, *p* = 0.04) and cisplatin/carboplatin group (HR 1.03, 95%CI 1.00–1.07, *p* = 0.04). PTEN-expression did not correlate with OS/DFS/LRC. Better organoid response to everolimus correlated significantly to higher p-mTOR expression (Rs = − 0.731, *p* = 0.04).

**Conclusions:**

High p-mTOR expression predicts and high p-ERK expression tends to predict worse treatment outcome in HPV negative HNSCC patients treated with chemoradiation, providing additional evidence that these markers are candidate prognostic biomarkers for survival in this patient population. Also this study shows that the use of HNSCC organoids for biomarker research has potential. The role of PTEN expression as prognostic biomarker remains unclear, as consistent evidence on its prognostic and predictive value is lacking.

**Supplementary Information:**

The online version contains supplementary material available at 10.1007/s12105-023-01576-4.

## Introduction

Head and neck cancer is the 7th most common type of cancer worldwide with approximately 1.000.000 new cases in 2020 [1]. Over 95% of these cancers are squamous cell carcinoma (SCC) [2–4]. In current practice, therapy for head and neck squamous cell carcinoma (HNSCC) generally depends on the anatomical location. For oral SCC, the standard treatment is primary surgery, whereas pharyngeal and laryngeal SCC often undergo primary (chemo)radiation. At the time of diagnosis, HNSCC has frequently spread to regional lymph nodes. Despite advances in surgical techniques and adjuvant therapies, survival rates have only marginally improved over the last two decades [5]. Therefore, there is a need for biomarkers predicting long-time survival that can help to guide treatment decisions and might lead to the development of new therapies [6, 7].

The phosphatidylinositol 3-kinase (PI3K)/AKT/mTOR signaling pathway is the most frequently altered pathway in HNSCC [8]. Normal activation of this signaling pathway fosters cell growth, survival, development and differentiation [9]. In HNSCC, genes in the PI3K-pathway are often mutated, amplificated and overexpressed causing aberrant signaling [10]. This affects normal cell growth, survival and differentiation contributing to development and maintenance of cancer. Numerous genetic alterations of upstream and downstream factors have currently been clarified. However, their predictive value has yet to be established.

The ‘mammalian target of rapamycin’ (mTOR) is part of the PI3K/AKT/mTOR-pathway and is a serine/threonine kinase which mediates cellular homeostasis and growth [11, 12]. mTOR is activated by phosphorylation (p-mTOR). Aberrant mTOR signaling is commonly observed in cancer, making it an interesting therapeutic target. mTOR-inhibition as therapy for HNSCC was reviewed in several clinical trials. Tumor response improved after treatment with mTOR-inhibition in combination with other agents [13]. Increased activation of mTOR is associated with worse survival in several types of cancer [14–19]. Also for oral, tongue and esophageal SCC, mTOR-expression is associated with worse survival [20, 21].

Phosphatase and tensin homolog (PTEN) is a protein encoded by the PTEN tumor suppressor gene [22]. PTEN is a natural inhibitor of the PI3K pathway and thereby a tumor suppressor gene. Loss of PTEN results in PI3K/AKT/mTOR pathway overactivity. PTEN mutations have been described in several tumor types [23–26]. Loss of PTEN on protein level correlates with a worse prognosis in breast, prostate and lung cancers [27–29]. In HNSCC, PTEN mutations are present in 5–10% of the patients [30–32]. Also for tongue cancer, loss of PTEN on protein level is associated with worse survival [33, 34].

Apart from the PI3K/AKT/mTOR pathway, the Ras/Raf/MEK/ERK pathway also contributes to cell cycle proliferation. Phosphorylated ERK (p-ERK) phosphorylates cytoskeletal proteins, kinases and several transcriptional factors [35], leading to cellular survival, proliferation, differentiation and angiogenesis [36]. ERK expression correlated with worse survival in several types of cancer [37–41]. In nasopharyngeal carcinoma, high p-ERK expression correlates with worse survival [42].

In this study, we assess the predictive value of p-mTOR, p-ERK and PTEN expression in a cohort of patients with HPV-negative oropharyngeal, hypopharyngeal and laryngeal SCC who were treated with primary chemoradiotherapy. Moreover, we assess the p-mTOR expression and mTOR inhibition response by everolimus in a cohort of HNSCC organoids.

## Materials and Methods

### Patients and Clinical Data

This study uses a retrospective cohort of patients with HNSCC, treated at the University Medical Center (UMC) Utrecht described previously by de Ruiter et al. [43]. Inclusion criteria were: HPV-negative oropharyngeal, hypopharyngeal and laryngeal SCC (1), treated with radiotherapy and concomitant cisplatin, carboplatin or cetuximab with curative aim (2) whereof both clinical response data and tumor tissue was available (3). Exclusion criteria were: previous radiotherapy in the head and neck region, complete surgical resection of tumor, presence of distant metastases, presence of a prognosis-affecting double or prior malignancy. The following clinical data were collected: age, sex, tumor site, T and N stage.

All patients were treated with primary chemoradiotherapy. Standard treatment consisted of 35 fractions of 2 Gy (total 70 Gy) on both the primary tumor and positive lymph nodes. An elective total dose of 46–57.75 Gy on other lymph nodes, combined with 3 cycles of cisplatin or carboplatin administered intravenously every three weeks, or weekly cetuximab intravenously.

### Tissue Microarray Construction and Immunohistochemistry

Pre-treatment biopsies were collected from every patient and were formalin fixed and paraffin embedded (FFPE). To determine representative tumor regions, sections of the FFPE blocks were stained with hematoxylin and eosin and assessed by a head and neck pathologist (SW). From these tumor regions, three tissue cores of 0.6 mm were obtained from the FFPE blocks and collected in a tissue microarray (TMA). The TMA was constructed using an automated tissue microarray instrument as described before [44]. TMA tissue sections (4 µm) were immunohistochemically stained with antibodies for the following antigens: phospho-mTOR (Ser2448; 49F9; 1:300; Cell Signaling), phospho-MAPK (ERK1/2) (p42/44; D13.14.4E; 1:400; Cell Signaling), PTEN (138G6; 1:100; Cell signaling). All antibodies were visualized with 3,3′-diaminobenzidine (DAB) chromagen and hematoxylin was used for counterstaining. Like TMA’s, organoids were FFPE and stained for phosphor-mTOR as described above.

### HPV Detection

All patients in this study had a HPV-negative tumor. Tumors were stained for p16 INK4a by immunohistochemistry (JC8, 1:1200, Immunologic) and considered HPV-negative if less than 70% of tumor cells stained positive. Presence of HPV-DNA was tested in p16 positive tumors by PCR. These tumors were excluded if high-risk HPV-DNA was detected [45].

### Immunohistochemical Analysis

The staining assessment was performed by a head and neck researcher (EDR) and a dedicated head and neck pathologist (SW), who were blinded for clinical outcome. Discrepancies were resolved by consensus. For each marker, staining intensity (0–3) and the percentage of stained tumor cells (0–100%) were scored. Cells were considered positive for p-mTOR and PTEN if expression was observed in the cytoplasm. Scoring of p-ERK was based on expression in the cytoplasm and in the nucleus. For organoids, expression of p-mTOR was scored in the same way as described above.

### Organoids and Everolimus

HNSCC organoids from oral cavity and larynx were cultured as described earlier by Driehuis et al. [46] and Millen et al. [47] Organoids were treated with everolimus (LC Laboratories, catalog no. E4040) an mTOR inhibitor. This drugscreen was described in detail by Driehuis et al. previously [46]. The Biobank Research Ethics Committee of the University Medical Center Utrecht (TCBio) approved the biobanking protocol: 12-093 HUB-Cancer according to the University Medical Center Utrecht (UMCU) Biobanking Regulation. All donors participating in this study signed informed-consent forms and can withdraw their consent at any time.

### Statistical Analysis

For each TMA core, a H-score was calculated by multiplying the intensity with the percentage of positive cells resulting in scores ranging from 0 to 300 [48]. The H-score for each tumor was calculated by averaging their corresponding TMA cores. Tumors were excluded from analysis if less than two TMA cores were assessable. Of patients where three TMA cores were available, intraclass correlation coefficients (ICCs) were calculated. A model with two-way mixed-effects was used [49].

The expression p-mTOR, p-ERK and PTEN was correlated to overall survival (OS), disease-free survival (DFS) and locoregional control (LRC). OS was defined as the number of days between the date of inclusion (first hospital visit before start of treatment) and date of death. DFS and LRC were defined as the number of days between the last day of radiotherapy and recurrence of the disease or date of death. Patients were censored at the date of last visit in case of absence of an event.

**Fig. 1:**
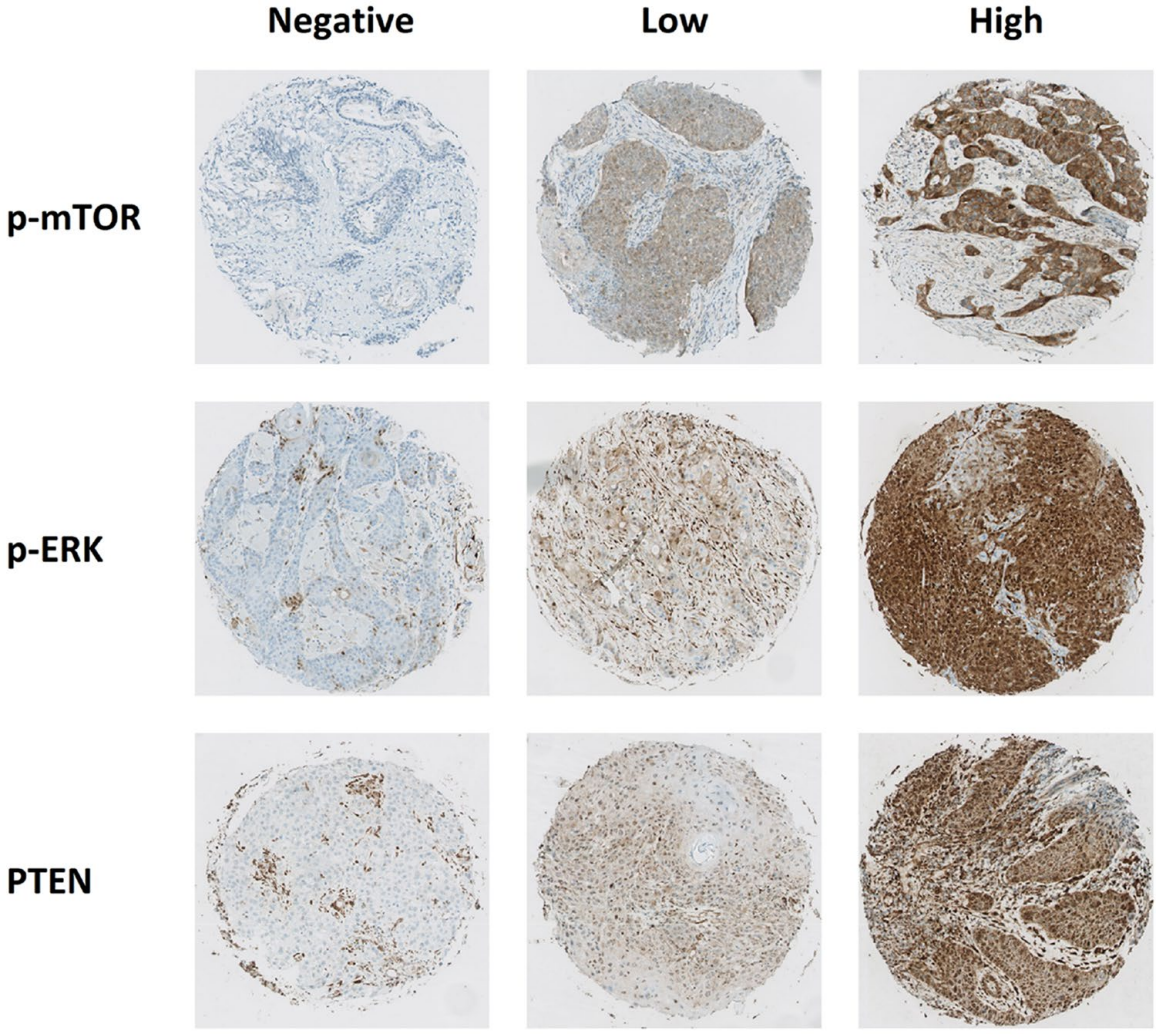
Representative images of TMA cores with negative, low and high scores for p-mTOR, p-ERK and PTEN expression

**Fig. 2:**
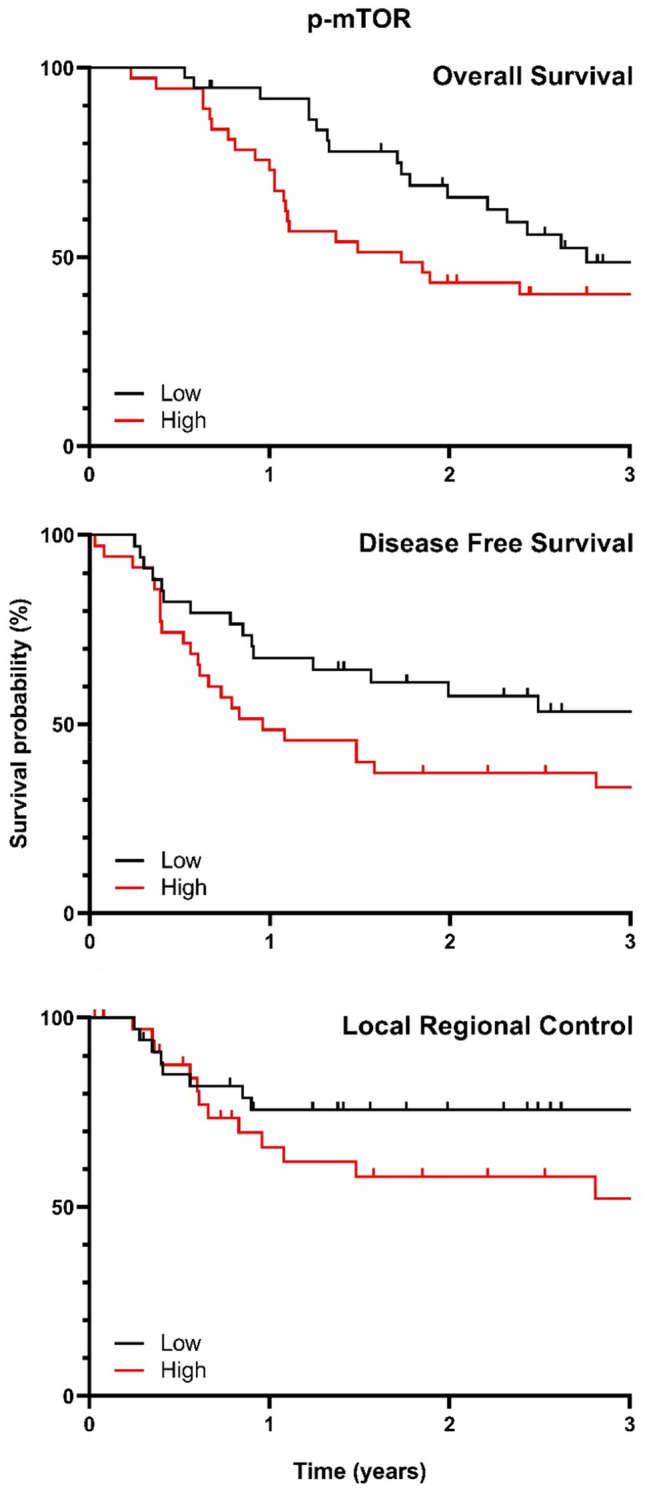
Kaplan–Meier curves visualizing the association between p-mTOR expression and OS, DFS and LRC. The median score of expression was used as cutoff for the survival analysis

Expression of biomarkers was correlated with clinical variables. The correlation between tumor site and biomarker expression was assessed using a Kruskal–Wallis test. For dichotomous variables and biomarker expression Mann–Whitney *U* tests were used. Correlation of age and biomarker expression was assessed with a Spearman’s rank correlation coefficient (Fig. 1). Univariate and multivariate cox proportional hazard regressions were used to assess the correlation of biomarker expression with OS, DFS and LRC. The multivariate model contained: biomarker as predictor corrected for age, gender, T-stage and N-stage. Statistical analysis was performed with SPSS Statistics (IBM Corp. Released 2020. IBM SPSS Statistics for Windows, Version 27.0. Armonk, NY: IBM Corp). The prognostic value of p-mTOR, p-ERK and PTEN expression was visualized by Kaplan–Meier curves comparing tumors with high and low biomarker expression stratified by the median value (Fig. 2, Supplementary Figs. 2, 3). A spearman’s rank-order correlation was run to assess the Correlation between p-mTOR and PTEN.

**Fig. 3:**
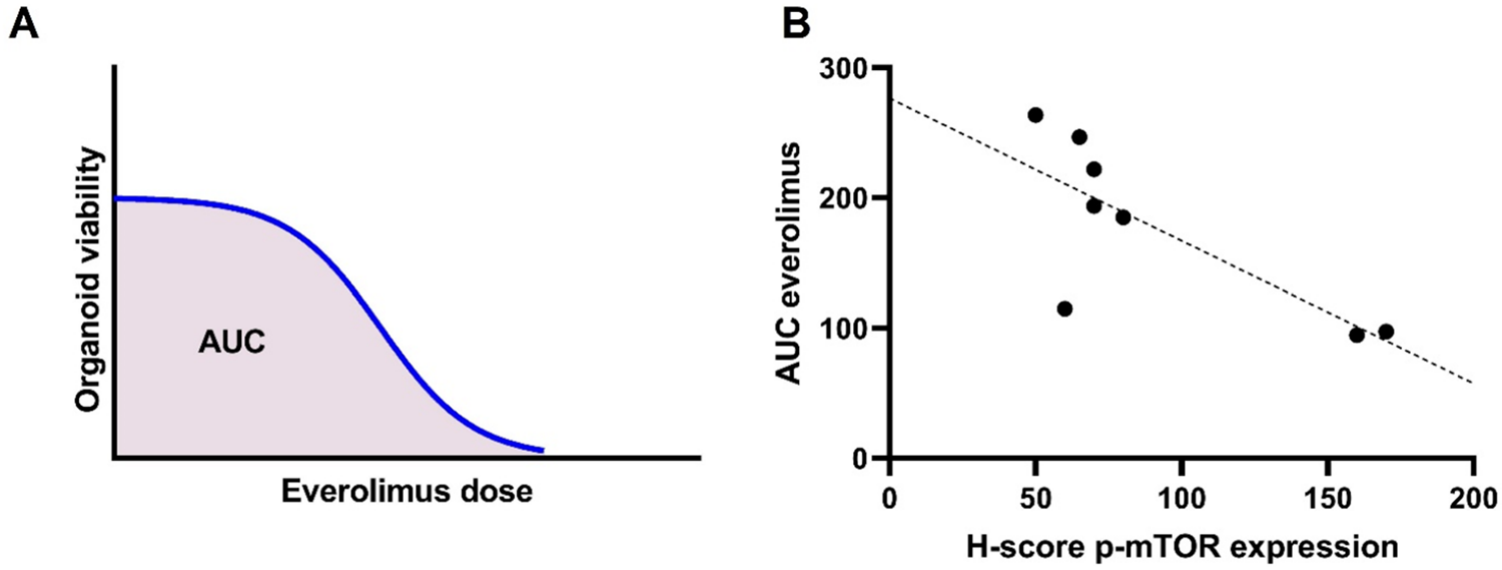
**A** Example of an everolimus dose–response curve depicted in blue with corresponding Area Under the Curve (AUC) shown in red. Everolimus dose increases on x-axis to the right. Organoid viability decreases if value on *Y*-axis is lower. **B** correlation of organoid response to everolimus (*Y*-axis) to H-score p-mTOR expression (*X*-axis)

For organoids a H-score of p-mTOR was calculated as described above. Response to everolimus was extracted using the Area Under the Curve (AUC) in the dose–response curve (Fig. 3). A spearman’s rank-order correlation was run to assess the relationship between H-score of p-mTOR expression and AUC of everolimus response in all HNSCC organoids.

## Results

### Patient Characteristics

A total of 77 patients with a mean age of 61.4 years were included for analysis: 48 with oropharyngeal, 16 with hypopharyngeal and 13 with laryngeal cancer. The 3- and 5-year OS of the patient cohort was 46% and 31%, respectively. The patient characteristics are described in Table 1.

**Table 1:** Characteristics study population

Characteristic	*n*	% of total *n*
Sex		
Female	31	(40.3%)
Male	46	(59.7%)
Tumor site		
Oropharynx	48	(62.3%)
Hypopharynx	16	(20.8%)
Larynx	13	(16.9%)
T-stage		
T1	1	(1.3%)
T2	4	(5.2%)
T3	27	(35.1%)
T4a	38	(49.4%)
T4b	7	(9.1%)
N-stage		
N0	9	(11.7%)
N1	6	(7.8%)
N2a	2	(2.6%)
N2b	23	(29.9%)
N2c	37	(48.1%)
Immunohistochemistry		
p-mTOR	75	(97.4%)
p-ERK	69	(89.6%)
PTEN	72	(93.5%)

All patients were treated with radiotherapy in combination with cisplatin (*n* = 53), carboplatin (*n* = 3) or cetuximab (*n* = 21).

A total of 8 HNSCC organoids were established including oral cavity (*n* = 6), larynx (*n* = 2).

### Immunohistochemistry

Of each tumor, three tissue cores were included in the TMA. However, some tissue cores were lost during processing or didn’t contain sufficient tumor cells. Cases were excluded if there were less than 2 out of 3 cores assessable. Consequently, for p-mTOR, 75 cases were eligible for inclusion, for p-ERK 69 cases, and for PTEN 72 cases.

Representative images of TMA cores containing low and high expression of each marker are displayed in Fig. 1. Boxplots were generated to represent the distribution of the scoring data of each marker (Supplementary Fig. 1). The median H-scores per marker of the patient cohort and organoids with corresponding interquartile ranges are displayed in Supplementary Table 1.

ICCs were calculated for three TMA cores from the same patients. Three TMA cores were available for p-mTOR (*n* = 57, 76%), p-ERK (*n* = 48, 70%) and PTEN (*n* = 51, 71%). ICC for p-mTOR cases was 0.83 (95%CI 0.73–0.89), for p-ERK cases 0.78 (95%CI 0.65–0.87) and for PTEN cases 0.88 (95%CI 0.81–0.93). ICCs for all three markers are therefore considered good [49].

### Correlation Clinicopathologic Parameters

No significant correlations between clinicopathologic parameters and immunohistochemical expression of p-mTOR, p-ERK and PTEN were observed. The results of all correlations are displayed in Supplementary Table 2. None of the clinical variables showed a correlation with OS, DFS and LRC (Table 2).

**Table 2:** Univariate/Multivariate analysis between markers and OS, DFS and LRC in whole study population

Univariate analysis			OS		DFS		LRC	
Marker	Comparison	*n*	HR (95%CI)	*p* value	HR (95%CI)	*p* value	HR (95%CI)	*p* value
p-mTOR	Per 10 H-score increase	75	**1.06 (1.01–1.11)**	**0.03**	1.04 (0.99–1.08)	0.11	1.00 (0.94–1.07)	0.98
p-ERK	Per 10 H-score increase	69	1.02 (0.99–1.05)	0.17	**1.03 (1.00–1.05)**	**0.04**	1.03 (1.00–1.06)	0.08
PTEN	Per 10 H-score increase	72	1.06 (0.99–1.15)	0.11	1.02 (0.95–1.10)	0.58	0.97 (0.88–1.07)	0.53
T stage	T1-3 vs T4	77	0.71 (0.38–1.32)	0.28	0.89 (0.50–1.57)	0.69	0.97 (0.46–2.05)	0.93
N stage	N0-1 vs N2-3	77	0.94 (0.44–2.03)	0.88	0.71 (0.33–1.51)	0.37	0.61 (0.21–1.75)	0.35
Age	Per 1 increase (year)	77	1.00 (0.95–1.05)	1.00	0.98 (0.93–1.02)	0.31	1.00 (0.94–1.06)	0.91
Sex	Male vs Female	77	1.43 (0.78–2.63)	0.25	1.55 (0.86–2.78)	0.14	2.07 (0.91–4.70)	0.08

Multivariate analysis			OS		DFS		LRC	
Marker	Comparison	*n*	HR (95%CI)	*p* value	HR (95%CI)	*p* value	HR (95%CI)	*p* value
p-mTOR	Per 10 H-score increase	75	**1.06 (1.01–1.11)**	**0.03**	1.04 (0.99–1.09)	0.11	1.00 (0.94–1.07)	0.96
p-ERK	Per 10 H-score increase	69	1.02 (0.99–1.05)	0.19	1.02 (0.99–1.05)	0.07	1.03 (0.99–1.06)	0.13
PTEN	Per 10 H-score increase	72	1.07 (0.99–1.15)	0.09	1.02 (0.95–1.10)	0.54	0.98 (0.88–1.08)	0.62

Univariate/Multivariate Cox proportional hazards regression of markers/clinicopathological parameters and overall survival (OS), Disease free survival (DFS) and Locoregional control (LRC). The prognostic values are displayed in Hazard Ratios (HR). 95%CI, 95% Confidence interval. Significant *p* values (*p* < 0.05) are shown in bold. Multivariate: Model contains biomarker as predictor corrected for age, gender, T-stage and N-stage

### p-mTOR Expression

A 10-point p-mTOR H-score increase correlated significantly with a worse OS in univariate analysis (HR 1.06, 95%CI 1.01–1.11, *p* = 0.03) (Table 2), sub analysis of the Cisplatin/Carboplatin group showed this worse OS likewise (HR 1.08, 95%CI 1.01–1.15, *p* = 0.02) (Table 3). In multivariate analysis a 10-point p-mTOR H-score increase remained independently correlated with worse OS in the whole patient cohort (HR 1.06, 95%CI 1.01–1.11, *p* = 0.03) (Table 2) as well as in the cisplatin/carboplatin group (HR 1.09, 95%CI 1.02–1.16, *p* = 0.02) (Table 3).

**Table 3:** Univariate/Multivariate sub-analysis between markers and OS, DFS and LRC in HPV negative patients treated with cisplatin/carboplatin

Univariate analysis			OS		DFS		LRC	
Marker	Comparison	*n*	HR (95%CI)	*p* value	HR (95%CI)	*p* value	HR (95%CI)	*p* value
p-mTOR	Per 10 H-score increase	54	**1.08 (1.01–1.15)**	**0.02**	1.06 (1.00–1.12)	0.06	1.02 (0.95–1.10)	0.55
p-ERK	Per 10 H-score increase	50	1.03 (0.99–1.06)	0.16	**1.03 (1.00–1.07)**	**0.04**	1.03 (1.00–1.08)	0.08
PTEN	Per 10 H-score increase	53	1.09 (0.99–1.20)	0.07	1.03 (0.94–1.13)	0.52	0.96 (0.85–1.10)	0.57
T stage	T1-3 vs T4	56	0.82 (0.39–1.72)	0.59	0.94 (0.47–1.86)	0.85	0.78 (0.32–1.93)	0.59
N stage	N0-1 vs N2-3	56	0.78 (0.27–2.24)	0.64	0.54 (0.19–1.53)	0.24	0.23 (0.03–1.69)	0.15
Age	Per 1 increase (year)	56	0.97 (0.91–1.02)	0.23	0.95 (0.90–1.00)	0.06	0.96 (0.89–1.03)	0.22
Sex	Male vs Female	56	1.10 (0.53–2.29)	0.80	1.22 (0.61–2.46)	0.57	1.47 (0.58–3.73)	0.42

Multivariate analysis			OS		DFS		LRC	
Marker	Comparison	*n*	HR (95%CI)	*p* value	HR (95%CI)	*p* value	HR (95%CI)	*p* value
p-mTOR	Per 10 H-score increase	54	**1.09 (1.02–1.16)**	**0.02**	**1.06 (1.00–1.12)**	**0.04**	1.02 (0.95–1.10)	0.54
p-ERK	Per 10 H-score increase	50	1.03 (0.99–1.07)	0.17	1.03 (1.00–1.06)	0.08	1.02 (0.98–1.06)	0.31
PTEN	Per 10 H-score increase	53	1.10 (1.00–1.21)	0.06	1.04 (0.94–1.14)	0.47	0.97 (0.86–1.10)	0.68

Univariate/Multivariate Cox proportional hazards regression of markers/clinicopathological parameters and overall survival (OS), Disease free survival (DFS) and Locoregional control (LRC). The prognostic values are displayed in Hazard Ratios (HR). 95%CI, 95% Confidence interval. Significant *p* values (*p* < 0.05) are shown in bold. Multivariate: Model contains biomarker as predictor corrected for age, gender, T-stage and N-stage

10-point p-mTOR H-score increase did not correlate with DFS and LRC in univariate and multivariate analysis in the patient cohort (Table 2). However, sub analysis of the cisplatin/carboplatin group showed a significant correlation with worse DFS in multivariate analysis (HR 1.06, 95%CI 1.00–1.12, *p* = 0.04) (Table 3). The prognostic value of p-mTOR in a Kaplan–Meier curve is displayed in Fig. 2. Sub analysis per subsite is displayed in Supplementary Table 3.

### p-ERK Expression

10-point p-ERK H-score increase correlated significantly with worse DFS in univariate analysis in the patient cohort (HR 1.03, 95%CI 1.00–1.05, *p* = 0.04 (Table 2) and in the cisplatin/carboplatin group (HR 1.03, 95%CI 1.00–1.07, *p* = 0.04) (Table 3). In multivariate analysis 10-point p-ERK H-score increase did not remain independently correlated with DFS, although trends were observed: whole patient cohort (HR 1.02, 95%CI 0.99–1.05, *p* = 0.07 (Table 2) and cisplatin/carboplatin group (HR 1.03, 95%CI 1.00–1.06, *p* = 0.08) (Table 3).

10 point p-ERK H-score increase did not correlate with OS and LRC in uni – and multivariate analysis. However trends were observed for worse LRC in univariate analysis; whole patient cohort (HR 1.03, 95%CI 1.00–1.06, *p* = 0.08) (Table 2) and cisplatin/carboplatin group (HR 1.03 95%CI 1.00–1.08, *p* = 0.08) (Table 3). The prognostic value of p-ERK in a Kaplan–Meier curve is displayed in Supplementary Fig. 2. Sub analysis per subsite is displayed in Supplementary Table 3.

### PTEN-Expression

10 point PTEN H-score increase did not correlate with OS, DFS and LRC. Though, a trend was observed in the cisplatin/carboplatin group for worse OS in univariate (HR 1.09, 95%CI 0.99–1.20, *p* = 0.07) (Table 3) and in multivariate analysis (HR 1.10, 95%CI 1.00–1.21, p = 0.06) (Table 3). The prognostic value of PTEN in a Kaplan–Meier curve is displayed in Supplementary Fig. 3. Sub analysis per subsite is displayed in Supplementary Table 3.

### Everolimus Organoid Response

The mean AUC of the organoids was 177.2 (95%CI 120.9–233.4). The mean H-score op p-mTOR expression in the organoids was 90.6 (95%CI 51.5–129.7). There was a statistically significant negative correlation between H-score and AUC Rs = − 0.731, *p* = 0.04 (Spearman’s rank order correlation), indicating a lower AUC (less organoid viability interpreted as better response to everolimus) correlates with a higher expression of p-mTOR measured as H-score.

## Discussion

HNSCC-survival only marginally improved over the last decades [5]. Therefore, there is a need for prognostic and predictive biomarkers for long-time survival that can help to guide treatment decisions and might lead to the development of new therapies [6, 7]. In this study we determined p-mTOR, PTEN and p-ERK expression and correlated it with survival in patients with HPV-negative oropharyngeal, hypopharyngeal and laryngeal SCC, treated with primary chemoradiotherapy and assessed p-mTOR expression and everolimus response in a subset of HNSCC organoids.

In solid tumors, mTOR is often activated [50]. The prognostic value of mTOR expression is still unclear. This study demonstrates in multivariate analysis that higher p-mTOR expression correlates with worse OS in a homogeneous cohort of 75 HNSCC patients. Several other studies also investigated the prognostic value of mTOR in HNSCC. For oral SCC, high p-mTOR expression correlated with poor survival in two studies [51, 52]. Li et al. investigated p-mTOR expression in patients with tongue SCC and found a worse overall survival with higher p-mTOR expression [21]. For laryngeal SCC, one study showed that high p-S6 expression, a surrogate marker of mTORC1 activation, correlated with improved survival [53]. This is not in line with the studies mentioned above, however, the investigated marker was different from p-mTOR. Although several subsites of HNSCC were examined, results are inconclusive.

Our study only included HPV-negative patients. HPV-positive patients are considered a separate group of HNCC and appear to have an increased survival [54, 55]. Wilson et al. assessed the prognostic value of mTOR in both HPV-negative and HPV-positive patients and found that high mTOR expression correlated with worse outcome in HPV-negative patients, which is in line with our findings. For HPV-positive patients they were not able to correlate mTOR expression with outcome [56]. This could be because HPV-positive patients harbor significantly less p53 mutations compared to HPV-negative patients [57] and, once activated, p53 is known to inhibit the activity of mTOR [58]. There are several studies that assessed the prognostic value of mTOR. Most of the studies included patients receiving postoperative chemotherapy and/or radiotherapy, which is different from our study that only included patients treated with primary chemoradiotherapy. The results of this study are in line with aforementioned studies [21, 51, 52], indicating that mTOR expression correlates with worse overall survival.

In addition to p-mTOR expression in patients, we investigated p-mTOR expression in a panel of HNSCC organoids. Here we show that HNSCC organoids express p-mTOR on protein level and that the expression, depicted in H-score, is comparable with p-mTOR expression in our patient cohort (Supplementary Fig. 1). As mTOR is a potential target for targeted therapy we assessed everolimus response in 8 HNSCC organoids and investigated if p-mTOR expression correlated with everolimus response. Despite the small sample size we demonstrated a correlation of p-mTOR expression and everolimus response. This finding underscores that organoid-platforms are suitable to perform biomarker research, validation and assessment of targeted therapies in HNSCC [46, 47].

As PTEN loss is more often seen in an aggressive tumor-type [59] it could explain the worse survival in HNSCC [33, 34]. Malfunctioning leads to overactivity of the AKT/mTOR signaling pathway, and a correlation between loss of PTEN and worse survival seems logical. However, the results of this study do not support this hypothesis. Patients with lower PTEN tumor expression even have a tendency to improved OS (*p* = 0.11). This could be explained by ignition of negative feedback loops by AKT/mTOR overexpression leading to higher PTEN expression in response [60]. In our cohort, sub analysis shows a statistically significant positive correlation between p-mTOR and PTEN expression Rs = 0.245, *p* = 0.04 which supports this hypothesis.

Lee et al. found worse survival in case of PTEN loss in patients with oral tongue SCCs receiving surgery [33]. Compared to this study, they investigated a different treatment modality and a different subsite. Additionally, PTEN expression can differ per subsite [61]. Also scoring systems of PTEN expression differ. Lee et al. compare PTEN expression in tumor and normal tissue to assess PTEN-loss [33]. Zhao et al. use four percentage categories of PTEN expressing cells combined with staining intensity [34] and our study uses a continuous scale for assessing the percentage of PTEN expressing cells multiplied by the staining intensity ranging from 0 to 3. These different scoring systems indicate a lack of standard approach. Furthermore, in literature, the terms ‘PTEN-expression’ and ‘PTEN-loss’ are used interchangeably and both arguments make it hard to compare results not supporting generalizability. In other tumor types, loss of PTEN expression has been linked to advanced stage disease [27–29] which in our study was not assessable as we only included advanced stage HNSCC. Overall, studies investigating PTEN expression report different findings for HNSCC.

The Ras/Raf/MEK/ERK pathway also contributes to cell cycle proliferation [35]. Activation results in cellular survival, proliferation, differentiation and angiogenesis [36]. P-ERK is one of the last steps in the pathway making it an interesting therapeutic target. ERK expression correlates with worse overall survival in several types of cancer [37–41].

For HNSCC, this study demonstrates that high p-ERK expression correlates with a worse DFS only in univariate analysis and not in the multivariate model (*p* = 0.07). The value of p-ERK as prognostic biomarker was analyzed in a few other studies. p-ERK expression correlated with a worse overall survival in nasopharyngeal carcinoma [42], esophageal SCC [62] and oral tongue SCC [63]. In contrast, the study of Psyrri et al. showed an improved overall survival with high ERK expression in patients with oral cavity, oropharyngeal, hypopharyngeal and laryngeal SCC [64]. Although this finding indicates the opposite, this correlation applies for the unphosphorylated ERK expression. Computational biology studies indicate that ERK is inversely correlated with p-ERK [65], meaning low levels of ERK correspond with high levels of p-ERK. With this in mind, the findings of Psyrri et al. [64] are in line with the aforementioned studies [42, 62, 63]. Since this study is partially in line with literature, p-ERK may be considered a possible prognostic biomarker for HNSCC.

A limitation of this study is the application of TMA’s that only partially represent the whole character of the tumor, which is inevitable in daily clinical practice after obtaining tissue biopsies. To overcome this issue, three TMA cores were taken per patient whereby the heterogeneity within the tumor biopsy is taken into account [66].

## Conclusion

This study shows that high p-mTOR expression predicts and p-ERK expression tends to predict worse treatment outcome in a cohort of advanced stage, HPV negative HNSCC patients treated with chemoradiation, providing additional evidence that these markers are candidate prognostic biomarkers for survival in this patient population. Also this study shows that the use of HNSCC organoids for biomarker research has potential. The role of PTEN expression as prognostic biomarker remains unclear, as consistent evidence on its prognostic and predictive value is lacking.

### Supplementary Information

Below is the link to the electronic supplementary material.

Supplementary file1 (DOCX 363 KB)

## Data Availability

The datasets generated during and/or analysed during the current study are available from the corresponding author on reasonable request.

## Code Availability

Not applicable.

### Stats Declaration

Statistical methods employed are performed by an experienced person, with authorship (acknowledgement) on the manuscript, and the contributor(s) specified.
