# Supplementary material for: p-mTOR, p-ERK and PTEN Expression in Tumor Biopsies and Organoids as Predictive Biomarkers for Patients with HPV Negative Head and Neck Cancer
Source: Head Neck Pathol. 2023 Jul 24;17(3):697–707. doi: 10.1007/s12105-023-01576-4 (PMC10514008; doi:10.1007/s12105-023-01576-4)
Supplement: Supplementary file 1 — Supplementary file1 (DOCX 363 KB) [file 12105_2023_1576_MOESM1_ESM.docx]

**Supplemental Table 1**

| **Patient cohort** | **Median score** | **Interquartile range** |
| --- | --- | --- |
| Marker  p-mTOR | 95 | 63 – 140 |
| p-ERK | 143 | 77 – 253 |
| PTEN | 60 | 41 – 85 |
| **Organoids** |  |  |
| Marker  p-MTOR | 70 | 38 - 103 |

**Supplemental Table 1:**  Median values of scoring per marker with corresponding interquartile ranges of patient cohort and organoids.

**Supplemental Table 2:** Correlations between immunohistochemical markers and clinical variables

|  | **p-mTOR** | **p** | **p-ERK** | **p** | **PTEN** | **p** |
| --- | --- | --- | --- | --- | --- | --- |
| **Age** | R=0.04 | 0.97 | R=0.07 | 0.59 | R=0.05 | 0.71 |
|  |  |  |  |  |  |  |
| **Sex**  Male  Female | 103 [63 – 147]  83 [63 – 120] | 0.51 | 137 [83 – 268]  148 [77 – 227] | 0.51 | 153 [90 – 280]  157 [77 – 227] | 0.44 |
|  |  |  |  |  |  |  |
| **Tumor site**  Oropharynx  Hypopharynx  Larynx | 103 [69 – 160]  80 [53 – 113]  98 [62 – 132] | 0.41 | 120 [57 – 270]  190 [82 – 310]  168 [127 – 220] | 0.58 | 130 [63 – 270]  195 [83 – 320]  167 [110 – 213] | 0.67 |
|  |  |  |  |  |  |  |
| **T stage**  T1-3  T4 | 100 [65 – 125]  91 [57 – 150] | 0.96 | 193 [93 – 258]  117 [58 – 208] | 0.09 | 203 [85 – 280]  120 [67 – 208] | 0.59 |
|  |  |  |  |  |  |  |
| **N stage**  N0-1  N2-3 | 117 [53 – 163]  87 [67 – 140] | 0.52 | 110 [50 – 208]  167 [83 – 258] | 0.24 | 112 [50 – 220]  167 [85 – 260] | 0.46 |
|  |  |  |  |  |  |  |

**Supplemental Table 2:** The correlation between markers and age was performed using Spearman Rho correlation. The correlation between markers and tumor site was performed using Kruskal-Wallis test. The other correlations were performed using Mann-Whitney U tests. Numbers displayed in table are as follows: Median, 25^th^ quartile, 75^th^ quartile and p-value.

| **Oropharynx treated with Cetuximab/Cisplatin/Carboplatin** | | | | | | | | |
| --- | --- | --- | --- | --- | --- | --- | --- | --- |
| **Univariate analysis** | | | **OS** | | **DFS** | | **LRC** | |
| **Marker** | **Comparison** | **n** | **HR (95%CI)** | **p-value** | **HR (95%CI)** | **p-value** | **HR (95%CI)** | **p-value** |
| p-mTOR | Per 10 H-score increase | 48 | 1.04 (0.98 – 1.09) | 0.22 | 1.02 (0.97 – 1.02) | 0.53 | 0.97 (0.90 – 1.04) | 0.39 |
| p-ERK | Per 10 H-score increase | 44 | 1.01 (0.98 – 1.05) | 0.57 | 1.01 (0.98 – 1.04) | 0.40 | 1.02 (0.98 – 1.05) | 0.42 |
| PTEN | Per 10 H-score increase | 43 | 1.08 (0.98 – 1.18) | 0.13 | 1.05 (0.96 – 1.14) | 0.34 | 0.98 (0.86 – 1.11) | 0.75 |
| T stage | T1-3 vs T4 | 48 | 0.54 (0.21 – 1.41) | 0.21 | 0.66 (0.29 – 1.53) | 0.34 | 0.70 (0.23 – 2.10) | 0.52 |
| N stage | N0-1 vs N2-3 | 48 | 1.22 (0.52 – 2.86) | 0.64 | 0.29 (0.68 – 3.62) | 0.29 | 2.17 (0.63 – 7.46) | 0.22 |
| Age | Per 1 increase (year) | 48 | 1.00 (0.93 – 1.07) | 0.94 | 0.99 (0.93 – 1.05) | 0.72 | 1.01 (0.93 – 1.09) | 0.85 |
| Sex | Male vs Female | 48 | 1.73 (0.83 – 3.63) | 0.14 | 0.57 (0.28 – 1.18) | 0.13 | 0.43 (0.16 – 1.20) | 0.11 |
| **Multivariate analysis** | | | **OS** | | **DFS** | | **LRC** | |
| **Marker** | **Comparison** | **n** | **HR (95%CI)** | **p-value** | **HR (95%CI)** | **p-value** | **HR (95%CI)** | **p-value** |
| p-mTOR | Per 10 H-score increase | 48 | 1.04 (0.98 – 1.10) | 0.16 | 1.02 (0.96 – 1.07) | 0.55 | 0.97 (0.89 – 1.05) | 0.39 |
| p-ERK | Per 10 H-score increase | 44 | 1.01 (0.98 – 1.05) | 0.59 | 1.01 (0.98 – 1.04) | 0.54 | 1.01 (0.97 – 1.05) | 0.67 |
| PTEN | Per 10 H-score increase | 43 | 1.10 (0.99 – 1.22) | 0.08 | 1.06 (0.96 – 1.16) | 0.28 | 0.99 (0.86 – 1.13) | 0.88 |
|  |  |  |  |  |  |  |  |  |
| **Larynx treated with Cetuximab/Cisplatin/Carboplatin** | | | | | | | | |
| **Univariate analysis** | |  | **OS** | | **DFS** | | **LRC** | |
| **Marker** | **Comparison** |  | **HR (95%CI)** | **p-value** | **HR (95%CI)** | **p-value** | **HR (95%CI)** | **p-value** |
| p-mTOR | Per 10 H-score increase | 12 | 1.21 (0.97 – 1.52) | 0.10 | 1.20 (0.98 – 1.46) | 0.07 | 1.19 (0.91 – 1.56) | 0.21 |
| p-ERK | Per 10 H-score increase | 12 | 1.19 (0.98 – 1.46) | 0.08 | 1.21 (0.98 – 1.50) | 0.07 | 1.36 (0.93 – 2.01) | 0.12 |
| PTEN | Per 10 H-score increase | 13 | 2.16 (1.00 – 4.67) | 0.05 | 1.15 (0.78 – 1.68) | 0.48 | 0.88 (0.59 – 1.31) | 0.52 |
| T stage | T1-3 vs T4 | 13 | 40.1 (0.01 - >999 | 0.37 | 40.6 (0.03 - >999 | 0.32 | 33.1 (0.01 - >999) | 0.53 |
| N stage | N0-1 vs N2-3 | 13 | 0.04 (0.00 - >999 | 0.59 | 0.04 (0.00 - >999) | 0.56 | 0.04 (0.00 - >999) | 0.72 |
| Age | Per 1 increase (year) | 13 | 1.04 (0.89 – 1.22) | 0.61 | 0.97 (0.86 – 1.11) | 0.69 | 0.97 (0.83 – 1.13) | 0.66 |
| Sex | Male vs Female | 13 | 2.35 (0.39 – 14.3) | 0.35 | 1.26 (0.25 – 6.29) | 0.78 | 0.71 (0.06 – 7.86) | 0.78 |
| **Multivariate analysis** | |  | **OS** | | **DFS** | | **LRC** | |
| **Marker** | **Comparison** |  | **HR (95%CI)** | **p-value** | **HR (95%CI)** | **p-value** | **HR (95%CI)** | **p-value** |
| p-mTOR | Per 10 H-score increase | 12 | 1.14 (0.88 – 1.48) | 0.31 | 1.18 (0.93 – 1.49) | 0.17 | 1.17 (0.85 – 1.61) | 0.34 |
| p-ERK | Per 10 H-score increase | 12 | 1.10 (0.85 – 1.44) | 0.46 | 1.19 (0.89 – 1.58) | 0.24 | >999 (0.00 - >999) | 0.34 |
| PTEN | Per 10 H-score increase | 13 | 1.73 (0.91 – 3.28) | 0.09 | 1.10 (0.73 – 1.66) | 0.65 | 0.86 (0.47 – 1.57) | 0.63 |
|  |  |  |  |  |  |  |  |  |
| **Hypopharynx treated with Cetuximab/Cisplatin/Carboplatin** | | | | | | | | |
| **Univariate analysis** | | | **OS** | | **DFS** | | **LRC** | |
| **Marker** | **Comparison** |  | **HR (95%CI)** | **p-value** | **HR (95%CI)** | **p-value** | **HR (95%CI)** | **p-value** |
| p-mTOR | Per 10 H-score increase | 15 | 1.03 (0.87 – 1.20) | 0.76 | 1.03 (0.90 – 1.18) | 0.69 | 1.07 (0.91 – 1.25) | 0.41 |
| p-ERK | Per 10 H-score increase | 13 | 1.03 (0.97 – 1.09) | 0.39 | 1.05 (0.99 – 1.12) | 0.10 | 1.06 (0.99 – 1.14) | 0.10 |
| PTEN | Per 10 H-score increase | 16 | 0.94 (0.79 – 1.13) | 0.54 | 0.95 (0.81 – 1.10) | 0.48 | 0.95 (0.80 – 1.14) | 0.60 |
| T stage | T1-3 vs T4 | 16 | 3.35 (0.39 – 28.9) | 0.27 | 4.48 (0.55 – 36.6) | 0.16 | 3.11 (0.36 – 26.9) | 0.30 |
| N stage | N0-1 vs N2-3 | 16 | 1.62 (0.19 – 14.0) | 0.66 | 1.30 (0.16 – 10.7) | 0.81 | 1.78 (0.21 – 15.4) | 0.60 |
| Age | Per 1 increase (year) | 16 | 0.96 (0.86 – 1.06) | 0.40 | 0.94 (0.86 – 1.03) | 0.21 | 1,00 (0.88 – 1.15) | 0.96 |
| Sex | Male vs Female | 16 | 0.45 (0.09 – 2.34) | 0.34 | 1.90 (0.47 – 7.61) | 0.37 | 1.85 (0.34 – 10.1) | 0.48 |
| **Multivariate analysis** | | | **OS** | | **DFS** | | **LRC** | |
| **Marker** | **Comparison** |  | **HR (95%CI)** | **p-value** | **HR (95%CI)** | **p-value** | **HR (95%CI)** | **p-value** |
| p-mTOR | Per 10 H-score increase | 15 | 0.94 (0.76 – 1.17) | 0.57 | 0.95 (0.79 – 1.15) | 0.61 | 0.99 (0.80 – 1.22) | 0.90 |
| p-ERK | Per 10 H-score increase | 13 | 1.03 (0.93 – 1.13) | 0.62 | 1.04 (0.96 – 1.14) | 0.33 | 1.05 (0.94 – 1.17) | 0.43 |
| PTEN | Per 10 H-score increase | 16 | 0.72 (0.46 – 1.13) | 0.15 | 0.70 (0.46 – 1.06) | 0.09 | 0.72 (0.46 – 1.15) | 0.17 |

**Supplemental Table 3:** **Univariate/Multivariate sub-analysis between markers and OS,DFS and LRC split by subsite**

**Supplemental Table 3:** Univariate/Multivariate Cox proportional hazards regression of markers/clinicopathological parameters and overall survival (OS), Disease free survival (DFS) and Locoregional control (LRC). The prognostic values are displayed in Hazard Ratios (HR). 95%CI, 95% Confidence interval. Significant p-values (p < 0.05) are shown in bold. Multivariate: Model contains biomarker as predictor corrected for age, gender, T-stage and N-stage.


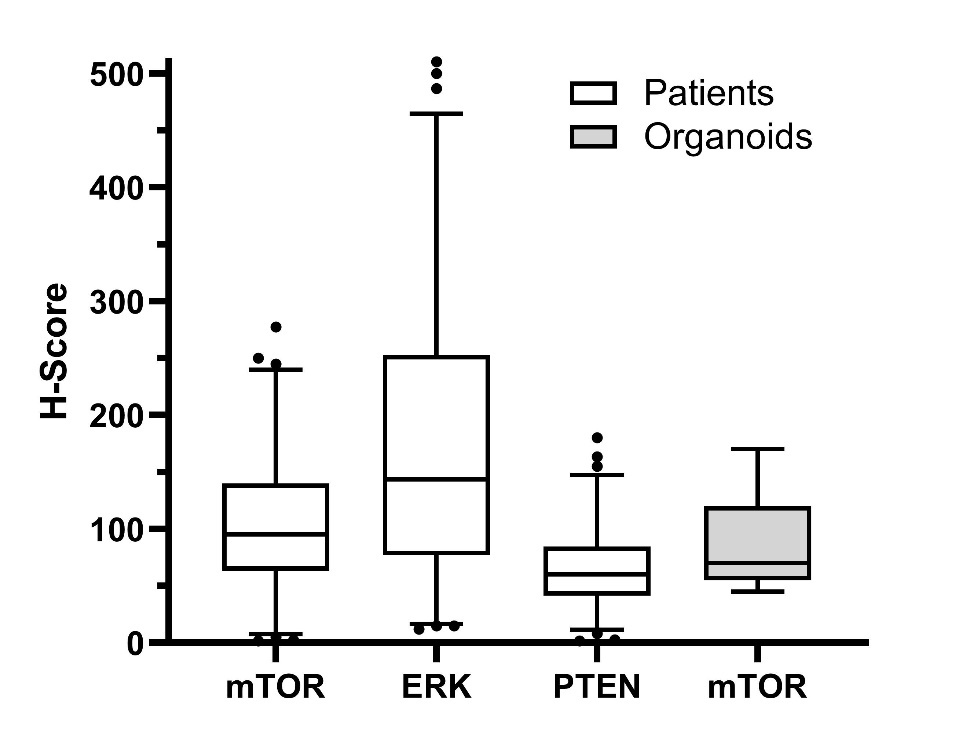


**Supplemental Figure 1:** Boxplot of H-score per marker (5-95%CI).


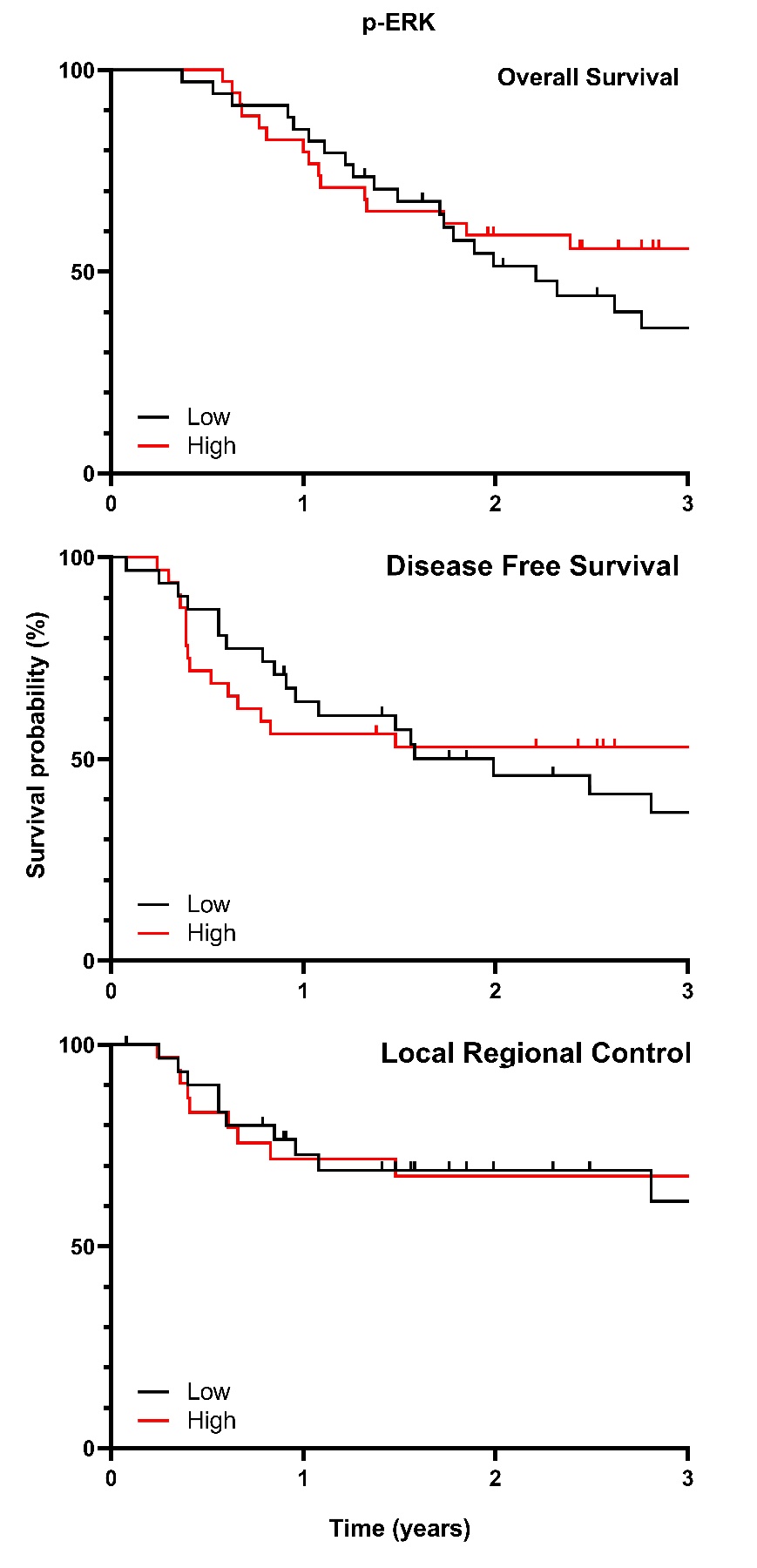


**Supplemental Figure 2:** Kaplan-Meier curves visualizing the association between p-ERK expression and OS, DFS and LRC. The median score of expression was used as cutoff for the survival analysis.


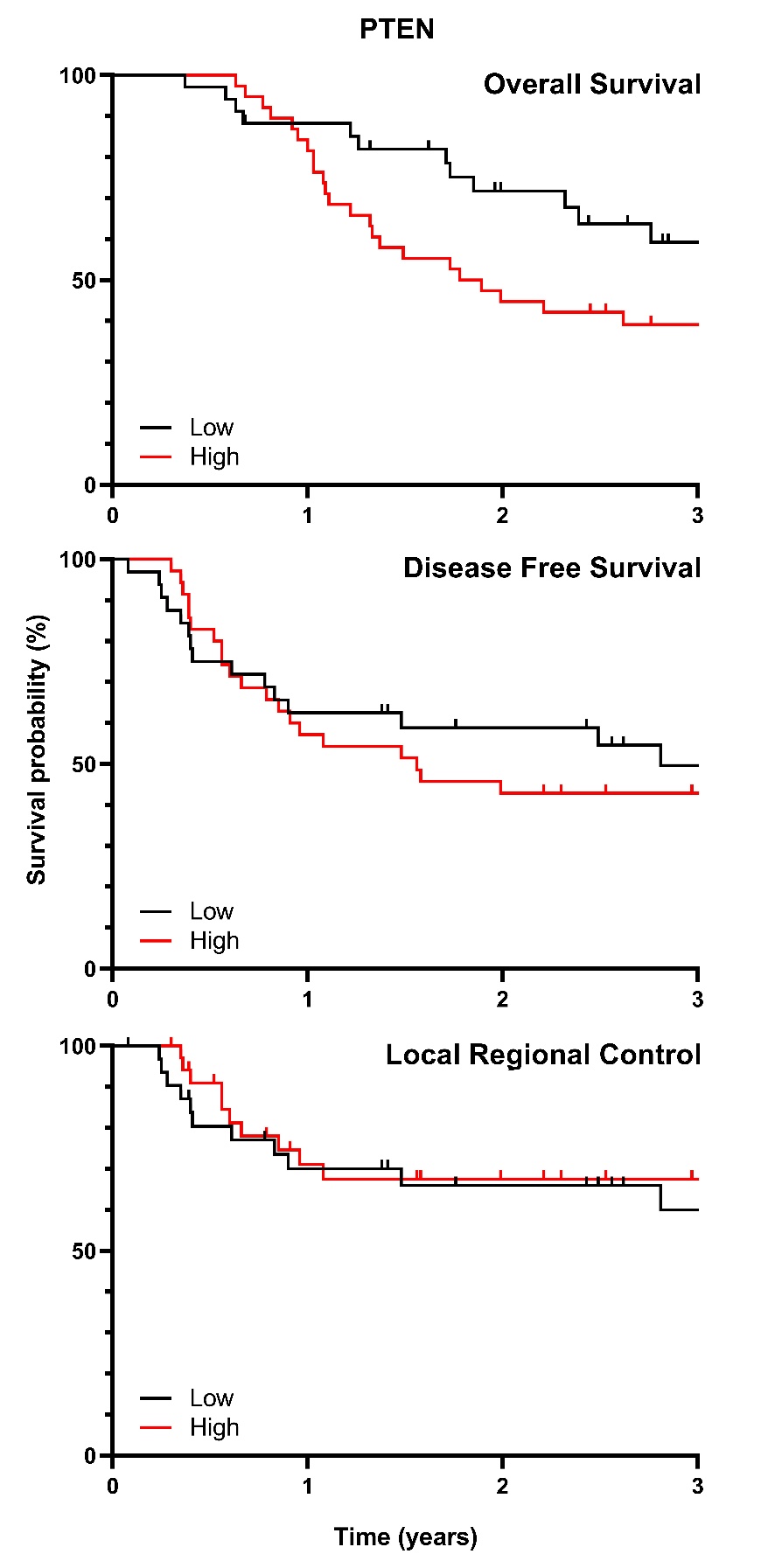


**Supplemental Figure 3:** Kaplan-Meier curves visualizing the association between PTEN expression and OS, DFS and LRC. The median score of expression was used as cutoff for the survival analysis.
